# Collagen I Microfiber Promotes Brain Capillary Network Formation in Three–Dimensional Blood–Brain Barrier Microphysiological Systems

**DOI:** 10.3390/biomedicines12112500

**Published:** 2024-10-31

**Authors:** Kimiko Nakayama-Kitamura, Yukari Shigemoto-Mogami, Marie Piantino, Yasuhiro Naka, Asuka Yamada, Shiro Kitano, Tomomi Furihata, Michiya Matsusaki, Kaoru Sato

**Affiliations:** 1 Laboratory of Neuropharmacology, Division of Pharmacology, National Institute of Health Sciences, 3-25-26, Tonomachi, Kawasaki-ku, Kawasaki City 210-9501, Kanagawa, Japan; k.kitamura@nihs.go.jp (K.N.-K.); shigemot@nihs.go.jp (Y.S.-M.); 2 Joint Research Laboratory for Social Implementation of Cultured Meat, Osaka University, 2-1 Yamadaoka, Suita 565-0871, Osaka, Japan; m-piantino@chem.eng.osaka-u.ac.jp (M.P.); m-matsus@chem.eng.osaka-u.ac.jp (M.M.); 3 Department of Applied Chemistry, Graduate School of Engineering, Osaka University, 2-1 Yamadaoka, Suita 565-0871, Osaka, Japan; yasuhiro-naka-st@tosoh.co.jp; 4 TOPPAN Holdings Inc., TOPPAN Technical Research Institute, 4-2 Takanodaiminami, Sugitomachi, Saitama 345-8508, Saitama, Japan; asuka.kato@toppan.co.jp (A.Y.); shiro.kitano@toppan.co.jp (S.K.); 5 Joint Research Laboratory (TOPPAN) for Advanced Cell Regulatory Chemistry, Graduate School of Engineering, Osaka University, Yamadaoka, Suita 565-0871, Osaka, Japan; 6 Laboratory of Advanced Drug Developmen Sciences, School of Pharmacy, Tokyo University of Pharmacy and Life Sciences, 1432-1 Horinouchi, Hachioji 192-0392, Tokyo, Japan; tomomif@toyaku.ac.jp

**Keywords:** blood–brain barrier (bbb), microphysiological system (MPS), collagen I microfiber (CMF), integrin, humanized model

## Abstract

Background: The blood–brain barrier (BBB) strictly regulates the penetration of substances into the brain, which, although important for maintaining brain homeostasis, may delay drug development because of the difficulties in predicting pharmacokinetics/pharmacodynamics (PKPD), toxicokinetics/toxicodynamics (TKTD), toxicity, safety, and efficacy in the central nervous system (CNS). Moreover, BBB functional proteins show species differences; therefore, humanized in vitro BBB models are urgently needed to improve the predictability of preclinical studies. Recently, international trends in the 3Rs in animal experiments and the approval of the FDA Modernization Act 2.0 have accelerated the application of microphysiological systems (MPSs) in preclinical studies, and in vitro BBB models have become synonymous with BBB–MPSs. Recently, we developed an industrialized humanized BBB–MPS, BBB–NET. In our previous report, we reproduced transferrin receptor (TfR)–mediated transcytosis with high efficiency and robustness, using hydrogels including fibrin and collagen I microfibers (CMFs). Methods: We investigated how adding CMFs to the fibrin gel benefits BBB-NETs. Results: We showed that CMFs accelerate capillary network formation and maturation by promoting astrocyte (AC) survival, and clarified that integrin β1 is involved in the mechanism of CMFs. Conclusions: Our data suggest that the quality control (QC) of CMFs is important for ensuring the stable production of BBB–NETs.

## 1. Introduction

The blood vessels in the central nervous system (CNS) limit the permeability of circulating substances into the brain via the blood–brain barrier (BBB) and protect the CNS from circulating xenobiotic compounds [1,2]. On the other hand, essential nutrients (such as glucose) are transported by specific transporters on vascular endothelial cells [3,4]. These functions of the BBB cause delays in drug development because it is difficult to predict the pharmacokinetics/pharmacodynamics (PKPD) [5,6], toxicokinetics/toxicodynamics (TKTD), toxicity, safety, and efficacy in the CNS [7,8]. In addition, BBB functional proteins show species differences [9,10]. Therefore, humanized in vitro BBB models are urgently needed to improve the predictability of preclinical studies [6]. In recent years, international trends in the 3Rs in animal experiments and the approval of the FDA Modernization Act 2.0 have accelerated the application of the microphysiological system (MPS) in preclinical studies. In vitro BBB models have recently become synonymous with BBB–MPS and are being developed in both industry and academia to respond to the BBB context of use (CoU).

**Figure 1:**
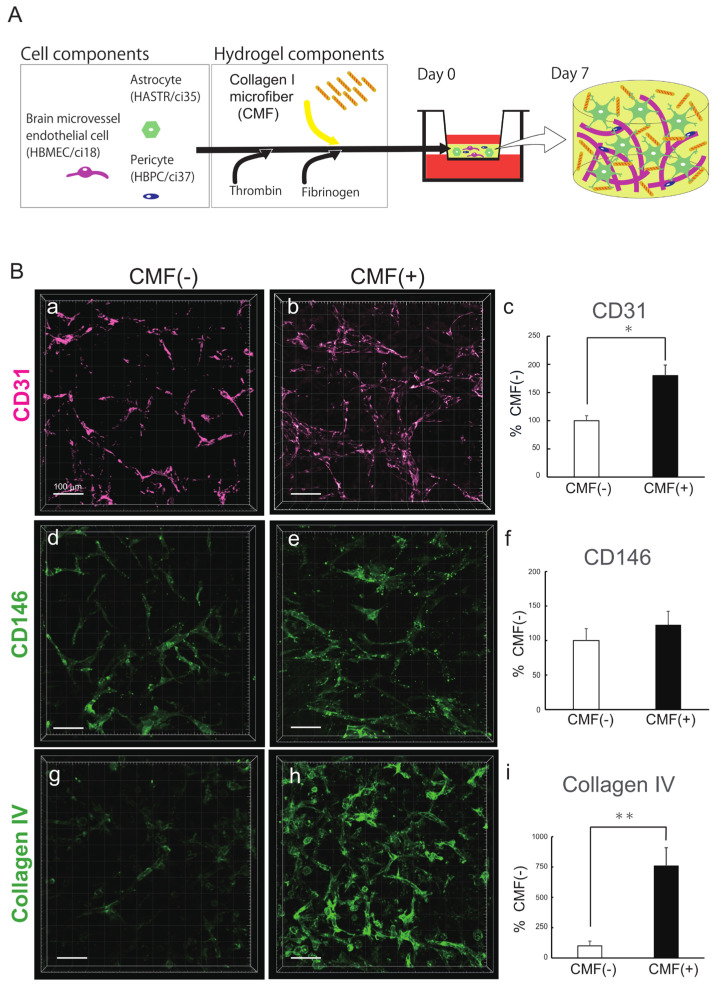
CMFs are important for the autonomous production of capillary networks. (**A**) Schematic of the BBB–NET components. (**B**) Representative z–stack images of the endothelial cell markers CD31 (**a**,**b**) and CD146 (**d**,**e**) and the basement membrane protein collagen IV (**g**,**h**) in hydrogels with (**b**,**e**,**h**) and without (**a**,**d**,**g**) CMFs. Scale bar: 100 μm. Comparison of the capillary volumes positive for CD31 (**c**), CD146 (**f**), and collagen IV (**i**) among hydrogels with and without CMFs. The percentage of positive staining in the presence of CMFs is shown, and without CMFs it was taken to be 100%. All data are expressed as the means ± standard errors of the means (SEMs). Each representation shown is from three independent experiments, each with four images. Student’s *t* test, *: *p* < 0.05, **: *p* < 0.01.

In vivo, the BBB mainly consists of endothelial cells (ECs), pericytes (PCs), basement membrane proteins, and astrocyte (AC) endfeet. Two categories of cell-based BBB-MPSs have been developed thus far: two-dimensional (2D) and three-dimensional (3D). In 2D BBB-MPSs, ECs, PCs, and ACs are arranged in a Transwell set in a multiwell plate. These models have been used for drug permeability tests for over 40 years [11,12,13]. Besides organoids, the first report of 3D BBB–MPSs was in 2017. Our group also developed 3D BBB-MPSs, in which immortalized human ECs (HBMECs/ci18), PCs (HBPCs/ci37), and ACs (HASTRs/ci35) self-organize to form a brain capillary network in hydrogel [14] (Figure 1A). The EC monolayers seeded on the bottom side of the hydrogel form opening structures that join the capillaries in the hydrogel. When drugs are placed outside the hydrogel, drug permeability can be assessed by measuring the amount of drug that permeates the BBB and moves into the supernatant above the hydrogel. We have succeeded in reproducing transferrin receptor (TfR)–mediated transcytosis in an efficient and robust manner [15]. We are now working on making this BBB–MPS into an industrial product, denoted as BBB–NET.

The BBB–NET hydrogels include fibrin and collagen I microfibers (CMFs). Each component has been reported to play important roles in vasculogenesis [16,17,18,19]. Shang F. et al. reported that the size and number of the opening structures on the bottom of the BBB–NET depend on the fibrin concentration. In a previous study, hard hydrogels (33 mg/mL fibrinogen) induced more active capillary organization than soft hydrogels with 7 mg/mL fibrinogen [20]. Collagen I has been used in tissue engineering and regenerative medicine [11,21], including in vitro BBB models [22], for several decades; however, the detailed effects of CMFs have not been confirmed. In this study, we investigated the advantages of using CMFs in addition to fibrin gel to fabricate BBB-NET. We showed that the CMFs accelerated the formation and maturation of the capillary network by promoting AC survival. We also determined that integrin β1 is involved in the CMF mechanism. Our data suggest that the quality control (QC) of CMFs is important for ensuring the stable production of BBB–NETs.

## 2. Materials and Methods

### 2.1. Materials

VascuLife basal medium (cat. no. LL-0002) and a VEGF LifeFactors kit (cat. no. LS–1020) were purchased from Kurabo (Osaka, Japan). Pericyte medium (cat. no. 1201) was purchased from ScienCell Research Laboratories (Carlsbad, CA, USA). Blasticidin S (cat. no. 029–18701) was purchased from Fujifilm Wako (Tokyo, Japan). Astrocyte growth medium (cat. no. A126130), fibronectin (cat. no. 33016015), anti–rabbit IgG conjugated with Alexa Fluor 488 (cat. no. A11031), anti–rabbit IgG conjugated with Alexa Fluor 647 (A21244), anti–rat IgG conjugated with Alexa Fluor 488 (cat. no. A11006), anti–mouse IgG conjugated with Alexa Fluor 488 (cat. no. A11029), anti–rabbit IgG conjugated with Alexa Fluor 647 (cat. no. A31571), and Cell Tracker (cat. no. C7025) were purchased from Thermo Fisher Scientific (Waltham, MA, USA). The anti–CD31 antibody (cat. no. 66065–2–1g) was obtained from Proteintech (Rosemont, IL, USA); the anti–CD146 antibody (cat. no. ab75769), anti–integrin β1 antibody (cat. no. ab24693), and mouse IgG, Kappa monoclonal, the isotype control (cat. no. ab91353), were purchased from Abcam (Cambridge, UK); and the anti–collagen IV antibody (cat. no. SGE–C–425) was purchased from COSMO BIO (Tokyo, Japan). The anti-aquaporin 4 antibody (cat. no. A5971), fibrinogen (F2006–5MG), and thrombin (T4648–10KU) were purchased from Merk (Darmstadt, Germany). DAPI (cat. no. 342–07431) was purchased from Dojindo (Tokyo, Japan). RapiClear 1.47 (cat. no. RC147001) and iSpacer (cat. no. IS002) were obtained from SunJin Laboratory (Hsinchu City, Taiwan, R.O.C.).

### 2.2. Cell Culture

Human brain microvascular endothelial cells/conditionally immortalized clone 18 (HBMECs/ci18), human brain pericytes/conditionally immortalized clone 37 (HBPCs/ci37), and human astrocytes/conditionally immortalized clone 35 (HASTRs/ci35) were established by Prof. Furihata T. et al. For maintenance, HBMECs/ci18 were grown in VascuLife complete medium, while HASTRs/ci35 and HBPCs/ci37 were cultured in astrocyte growth medium and pericyte medium, respectively. The composition of the medium is described in reference [23], and all culture media contained 4 mg/mL blasticidin S. The cells were cultured at 33 °C for growth and at 37 °C for differentiation.

### 2.3. CMF and BBB-NET Preparation

Collagen I microfibers (CMFs) were established by Prof. Matsusaki [24] and supplied by the Toppan Technical Research Institute. Gel preparation was described by Agathe F. et al. [13]. Briefly, 70 μL of gel containing 0.7 mg of CMFs was mixed with 0.4 mg of fibrinogen in 40 μL of DMEM in one tube, while in another tube, 2.0 × 10^5^ HBMECs/ci18, 4.0 × 10^5^ HASTRs/ci35, and 1.0 × 10^5^ HBPCs/ci37 were mixed with 0.3 U of thrombin in 30 μL of VascuLife complete medium. The solution was mixed just before being added to the top of a previously plasma–treated and fibronectin-coated culture insert. After 45 min of gelation, 2 mL of mixed media (1:1:1 complemented VascuLife/pericyte medium/astrocyte medium) was added. The gels were incubated for 7 days at 37 °C in a humidified atmosphere with 5% CO_2_, and the medium was changed every 3–4 days (Figure 1A).

### 2.4. Astrocyte (HASTR/ci35) Labeling

HASTRs/ci35 (1.0 × 10^6^ cells) were centrifuged, and the supernatant was aspirated. The cells were then gently resuspended in prewarmed Cell Tracker working solution (Thermo Fisher Scientific). After 45 min of incubation at 37 °C, the cells were centrifuged and the working solution was removed.

### 2.5. Blocking the CMF–Integrin Interaction in Astrocytes

HASTRs/ci35 (1.0 × 10^6^) were preincubated with an integrin β1–blocking mAb or isotype control [25,26,27], after which the gels were prepared as described previously. After 7 days of incubation at 37 °C with 5% CO_2_, the cells were stained with DAPI and counted.

### 2.6. Immunostaining

After 7 days of culture, the 3D BBB models were fixed with 4% paraformaldehyde (Fujifilm Wako, Osaka, Japan) for 20 min of incubation at room temperature, after which the cells were washed with PBS three times before 2 hr of incubation in blocking solution (10% normal goat serum and 0.2% Triton–X in PBS) at room temperature. The samples were incubated with primary antibodies for 2 days at 4 °C in 1% normal serum with 0.2% Triton X–100. The concentrations of the primary antibodies against the human proteins used were as follows: anti–CD31 (200×), anti–CD146 (200×), and anti–collagen IV (50×). The cells were then washed with 0.1% Triton–X in PBS and incubated with PBS containing secondary antibodies (anti-rabbit IgG conjugated with Alexa Fluor 488 or 647, anti–rat IgG conjugated with Alexa Fluor 488, or anti–mouse IgG conjugated with Alexa Fluor 488 or 647; each 500×) for 3 hr at room temperature. The samples were washed and counterstained with DAPI (1000×). They were then washed with PBS 3 times, and cleared with RapiClear according to the manufacturer’s instructions until they became transparent. The gels were removed and sandwiched between coverslips using iSpacer (Sunjin Laboratory, Hsinchu City, Taiwan.).

### 2.7. Analysis of Protein Expression in the 3D BBB Models

Fluorescence images were obtained using a Nikon A1R–A1 confocal microscope system (Nikon, Tokyo, Japan). Z–stack images were taken 30–80 μm from the bottom of the gel at 20× magnification. In one well, the coordinates of four fields of view were determined. Four images taken at the above coordinates per one well were taken from each well. To reconstruct the stained images as 3D images, the Surface tool in IMARIS software v. 9.8.2 (Oxford Instrumentals, Abingdon, UK) was used with the following four steps: Default Basic Algorithm, Source Channel, Threshold, and Classify Surface. The lowest threshold was set so that the target protein signal intensity could be differentiated from the background signal. Nonspecific signals were removed automatically by the Filter program in Classify Surface mode. The 3D rendered images were subsequently constructed, and representative images are shown in Figure S1. All the images were processed with the same parameters. The vessel volume was calculated and summed according to our previous methods [28].

### 2.8. Statistical Analysis

All the data are expressed as the means ± standard deviations unless otherwise noted. Statistical analyses were performed as shown in the figure legends, and *p* values less than 0.05 were considered to indicate statistical significance. The results were confirmed through 2–3 independent experiments.

## 3. Results

### 3.1. The Collagen I Microfibers in BBB–NET Hydrogel Enhanced Autonomous Brain Capillary Network Formation

To clarify the role of the CMFs in the BBB–NET in terms of capillary network formation, we first compared the capillary networks in the hydrogels made from fibrin alone (CMF [25,26,27]) with those made from fibrin and CMF (CMF [+]) (Figure 1A,B). The capillary volume in BBB–NET was quantified with IMARIS analysis of CD31–positive tubes. CD31 has long been used as a mature endothelial marker [29,30,31]. The CD31-positive capillary volume in the CMF (+) gel was significantly greater than that in the CMF (−) gel (Figure 1B, a–c), reaching 181.93 ± 16.3% that of the CMF (−) gel group. Because CD146 is expressed by immature endothelial cells [32], we also compared the CD146–positive capillary volumes between the CMF (−) group and the CMF (+) group (Figure 1B, d–f). The CD146-positive capillary volume in the CMF (+) gel tended to be greater than that in the CMF (−) gel (122.9 ± 19.4% that in the CMF (−) gel), but the difference was not significant. These results suggested that CMFs have a stronger effect on maturation than capillary formation. As capillaries mature, the vascular basement membrane develops, which contributes to BBB integrity. The vascular basement membrane forms a 3D protein network predominantly composed of collagen IV, laminin, nidogen, and heparan sulfate proteoglycans, which support BBB integrity and interactions among BBB cells [33]. We therefore compared the development of basement membrane in the CMF (−) gels and CMF (+) gels in terms of collagen type IV (Col IV) expression levels (Figure 1B, g–h). The volume of Col IV–positive capillaries in the CMF (+) gel was markedly greater than that in the CMF (−) gel (758.8 ± 150.8% of that in the CMF (−) gel group). These results suggest that CMFs enhance autonomous brain capillary network formation in BBB–NETs.

### 3.2. CMFs Supported Astrocyte (HASTRs/ci35) Survival

**Figure 2:**
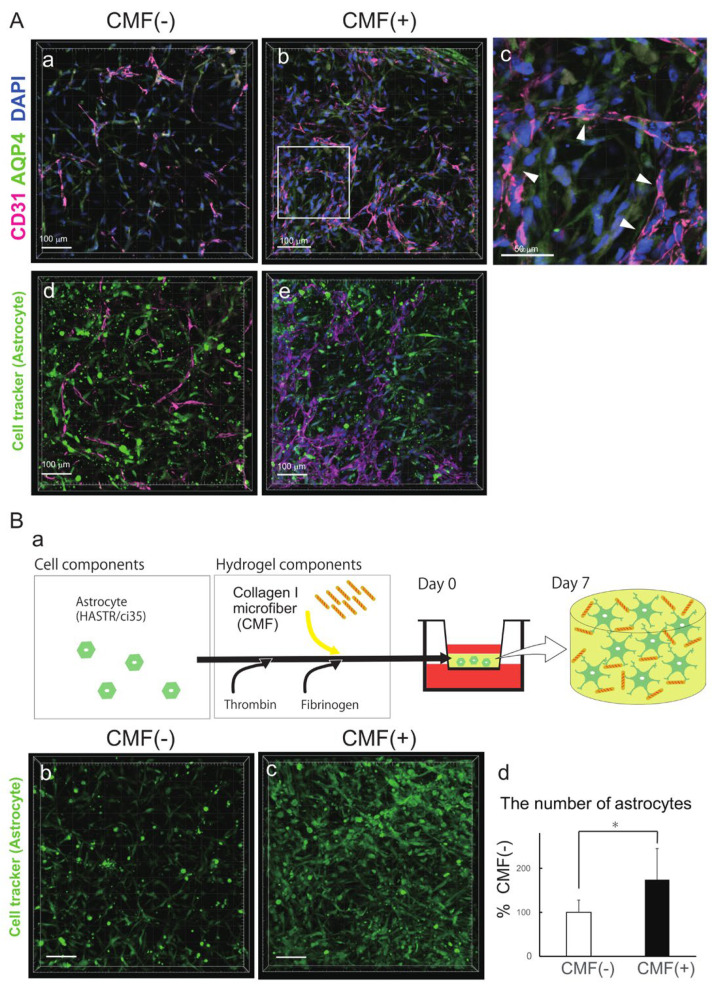
CMFs support astrocyte survival. (**A**) Representative z-stack images of the astrocyte marker aquaporin 4 (**a**–**c**) and Cell Tracker-labelled astrocytes (**d**,**e**) in hydrogels with (**b**,**c**,**e**) and without (**a**,**d**) CMFs. Arrows in (**c**) show AQP4–positive ACs surrounded CD31-positive capillaries. Scale bar in (**a**,**b**,**d**,**e**): 100 μm; scale bar in (**c**): 50 μm. (**B**) Schematic of the astrocyte−only gel (**a**). Representative z−stack images of Cell Tracker−labelled astrocytes in hydrogels with (**c**) and without (**b**) CMFs. Comparison of the number of astrocytes in hydrogels with and without CMFs (**d**). Each representation shown is from three independent experiments, each with four images. Student’s *t* test, *: *p* < 0.05. Scale bar in (**b**,**c**): 100 μm.

We found that there were more ACs in the CMF (+) hydrogel than in the CMF (−) hydrogel (Figure 2A). To examine the effects of the CMFs in more detail, we compared the ACs in the CMF (−) BBB–NET with those in the CMF (+) BBB–NET via aquaporin 4 (AQP4) immunostaining (Figure 2A, a–c) and Cell Tracker staining (Figure 2A, d,e). AQP4–positive ACs surrounded CD31-positive capillaries in the CMF (+) gel (Figure 2A, c). We therefore investigated the effect of the CMFs on the number of ACs (Figure 2B, a). We used Cell Tracker and DAPI for cell counting (Figure 2B, b–d) because immunostaining for AQP4 or GFAP does not reveal cell morphology due to the localization of these proteins. The number of astrocytes in the CMF (+) hydrogel group was significantly greater than that in the CMF (−) hydrogel group (Figure 2B, d); 174.1 ± 70.7% that of the CMF [32] group). These results showed that CMFs support AC survival in BBB–NETs.

### 3.3. Astrocytes Promote the Formation and Maturation of Capillary Networks

**Figure 3:**
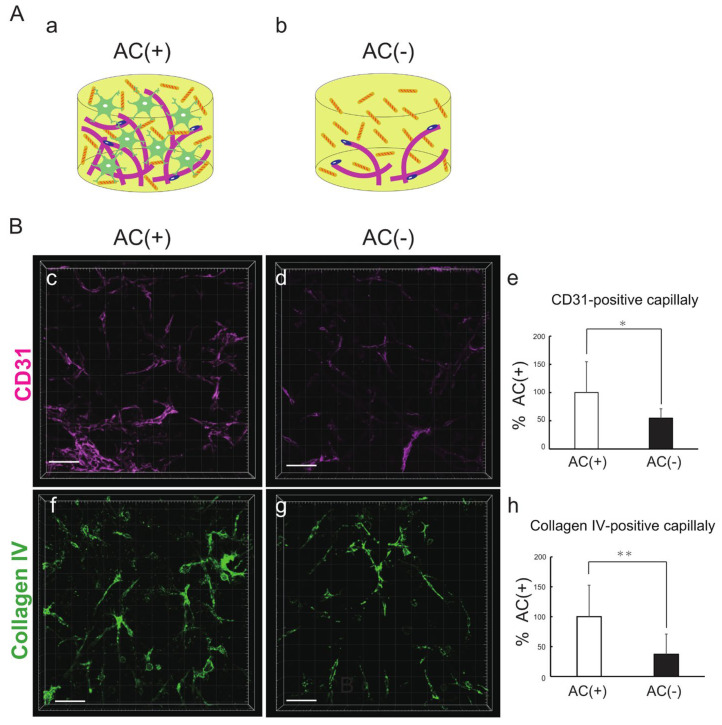
Astrocytes promote the formation and maturation of capillary networks. (**A**) Schematic of hydrogels with CMFs and with (**a**) and without (**b**) ACs. (**B**) Representative z–stack images of CD31 (**c**,**d**) and collagen IV (**f**,**g**) in the presence (**c**,**f**) and absence (**d**,**g**) of astrocytes in the hydrogel with CMFs. Scale bar: 100 μm. Comparison of CD31–positive capillary volume (**e**) and collagen IV–positive blood vessels (**h**) in the absence and presence of astrocytes in the hydrogel with CMFs. Each representation shown is from three independent experiments, each with four images. Student’s *t* test, *: *p* < 0.05; **: *p* < 0.01.

On the basis of the results described above, we speculated that an increase in the number of ACs would be advantageous for capillary network formation. We therefore generated BBB–NET with and without ACs (Figure 3A). After quantifying the CD31–positive capillary volume with IMARIS software (Figure 3B, c–e), it was revealed that the capillary volume significantly decreased when ACs were removed from the CMF (+) hydrogel (Figure 3B, e); 54.6 ± 16.7% that of the AC (+) group). We also examined the effects of ACs on capillary basement membrane formation by observing the expression of collagen IV, one of the major components of the basal membrane (Figure 3B, f–h). The volume of collagen IV–positive capillaries was significantly decreased when the ACs were removed (Figure 3B, h); 37.1 ± 33.9% that of the AC (+) group). In addition, the signal strengths of both CD31 and collagen IV increased. These results indicate that the positive effects of CMFs are mediated by an increase in AC survival, which promotes capillary formation and maturation.

### 3.4. CMFs Enhanced Astrocyte (HASTR/ci35) Survival by Interacting with Integrin β1

**Figure 4:**
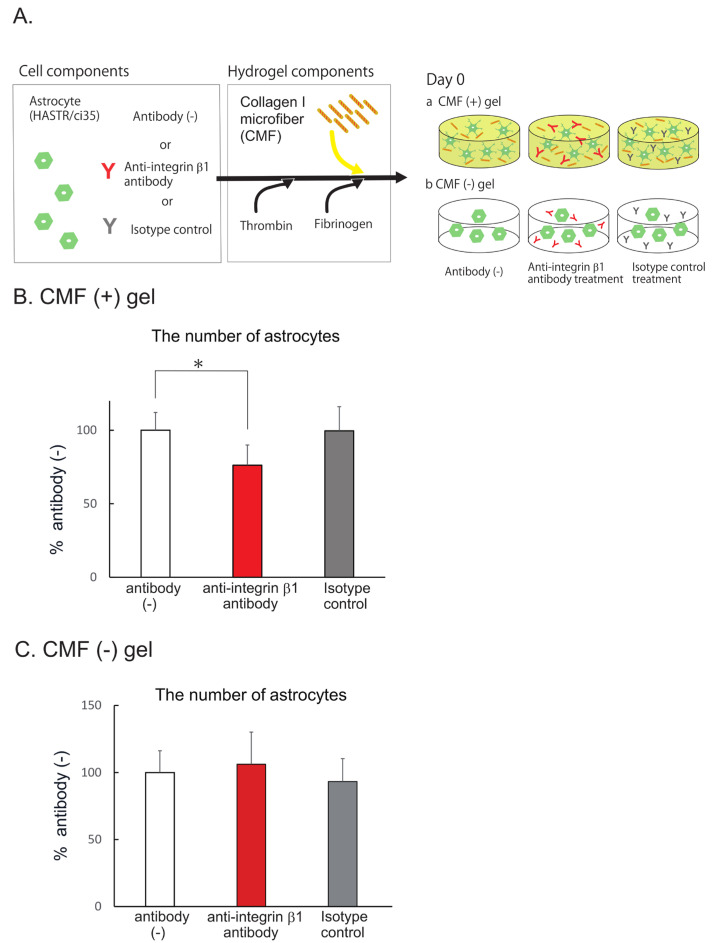
CMFs interact with astrocytes via the integrin β1 subunit. (**A**) Schematic of AC pretreated with and without each antibody in hydrogels with (**a**) and without CMF (**b**). (**B**) Comparison of the number of astrocytes after pretreatment with an integrin β1 antibody or an isotype control in hydrogels with CMFs. Each representation shown is from three independent experiments, each with four images. Student’s *t* test, *: *p* < 0.05. (**C**) Comparison of the number of astrocytes after pretreatment with an integrin β1 antibody or an isotype control in hydrogels without CMFs. Each representation shown is from two independent experiments, each with four images.

The CMFs used in this study are collagen I fragments with a size of 20 μm that crosslink with each other [24]. Collagen I has been reported to interact with several membrane proteins, such as integrins (α1β1, α2β1, α10β1, and α11β1), discoidin domain receptor (DDR) 1, DDR2, osteoclast-associated receptor (OSCAR), glycoprotein VI (GPVI), G6b–B, leukocyte-associated Ig-like receptor–1 (LAIR–1) of the leukocyte receptor complex (LRC), and the mannose family receptor uPARAP/Endo 180 [34]. Among these membrane proteins, the integrins and DDR1 are expressed in ACs [35], suggesting that they function as collagen receptors in our experiments. Integrins are active as heterodimers, and there are a wide variety of combinations of subunits [35]. When integrins function as collagen receptors, the β1 subunit is included [34]. We therefore examined the contribution of the CMF–integrin β1 interaction using an anti-integrin β1 antibody [27] or an isotype control (Figure 4A). After 7 days of AC coculture with the antibody, we counted the number of cells. In the anti-integrin β1 antibody-treated group, the number of ACs was significantly lower than that in the antibody (–) group (Figure 4B, 76.2 ± 13.8% that of the antibody (–) group), whereas the number of ACs in the isotype control group was almost the same as that in the antibody (–) group (99.6 ± 16.4% that of the antibody (–) group). The BBB–NET hydrogel contains fibrinogen, which contains an Arg–Gly–Asp (RGD) sequence that also binds to the integrin β1 subunit. We therefore confirmed the extent to what the interaction between fibrin and integrin β1 contributed to AC survival using the fibrin hydrogels (Figure 4C). In the hydrogel without CMFs, the anti–integrin β1 antibody did not affect the number of surviving cells. These data suggest that the CMFs interact with integrin β1 on ACs and promote their survival, and that increasing the number of ACs further promotes the formation of capillary networks in BBB–NETs.

## 4. Discussion

Our 3D–type BBB-MPS, BBB–NET, uses a hydrogel that includes fibrin and CMFs fabricated from collagen I [24]. This combination has long been used in the field of cell engineering because of its reasonable cost [15,16,17]. In vivo, brain capillaries are characterized by their unique basement membrane (BM) structure, which is composed of extracellular matrix (ECM) components [36], such as laminin [36,37], collagen IV [38,39], fibronectin [40], and heparan sulfate proteoglycans [41,42]. In addition, collagen IV is one of the major components of the BM [33,43], and collagen I has also been reported to be involved in the development of the CNS [42].

The CMFs used in this study were designed to be ≈20 μm, which is shorter than the cell body lengths of ECs and ACs [24]. This size enables the CMFs to have a sedimentation velocity similar to that of these cells because of their size ranges, thereby achieving homogeneous cell distribution in BBB-NETs. Our study revealed that CMFs promoted AC survival, thereby increasing capillary network formation. The data also suggest that the interaction between ECs and ACs is important for capillary self-organization. Furthermore, the increased number of ACs also upregulated the expression of CD31 and collagen IV. Originally, collagen I and fibrin were used as angiogenic polymers in tissue engineering [44], and CMFs have been reported to enhance the survival of human umbilical vein endothelial cells (HUVECs) [45]. Our data suggest that, in the presence of astrocytes, CMFs further enhance capillary maturation and capillary formation through the indirect effects of astrocytes. Astrocytes release many kinds of factors, including peptides, growth factors, and chemokines [46,47,48,49,50] used in the interaction with ECs, and their identification is our next focus.

In mammals, integrin heterodimers are composed of α and β subunits that form a noncovalent complex. To date, 18 α subunits and 8 β subunits have been identified, and 24 functionally distinct heterodimeric transmembrane receptors with different combinations of α and β subunits have been reported [34,35]. Among the integrin receptors, α1β1, α2β1, α10β1, and α11β1 bind to collagen I [35,51], and some amino acid sequences that are necessary for binding have been identified [such as Gly–Phe–Hyp–Gly–Glu–Arg (GFOGER)] [52]. Our data indicate that the effects of CMFs on astrocytes are partly mediated through interactions with integrin receptors, including integrin β1. CMFs have protective effects on AC survival through binding integrin receptors, including β1. Integrins transactivate growth factor receptors even in the absence of growth factor ligands and subsequently activate intracellular signal transduction cascades [53]. By binding to collagen or fibronectin, integrin β1 has also been reported to induce tyrosine phosphorylation on growth factor receptors, such as the EGF receptor [54], PDGFRβ [55], and vascular endothelial growth factor (VEGF) receptor–3 [56,57,58], in the absence of their respective ligands [33]. On the other hand, ACs have been reported to support angiogenesis through integrin β1 [58]. These previous studies support our hypothesis that CMFs promote the survival of ACs via the transactivation of cell surface growth factor receptors through the interaction with integrin β1, thereby promoting BBB–NET capillary network formation and maturation. Although the effects of the functional blocking antibody were significant, they remained at 76.2 ± 13.8% that of the control, suggesting that other mechanisms also contribute to the effects of CMFs. As described above, the collagen-binding integrins are α1β1, α2β1, α10β1, and α11β1. Further experiments are necessary to identify which α subunits are involved in the effects of the CMFs. In addition to integrins, DDR1 is another CMF candidate receptor. It has been reported that DDR1 interacts with collagen I [26] and is expressed in astrocytes [59,60]; however, little information is available concerning the role of DDR1 in angiogenesis. It will therefore be necessary to confirm whether or not the effects of the CMFs are mediated through the mechanisms described above in our next study.

## 5. Conclusions

**Figure 5:**
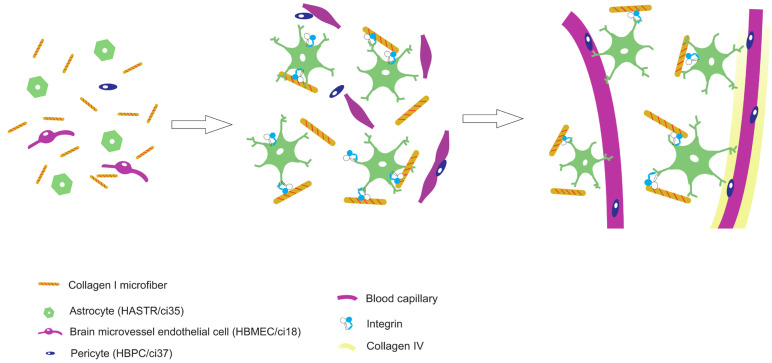
Our hypothesized mechanism by which CMFs promote capillary formation and maturation in BBB-NETs. CMFs increased the number of surviving ACs by interacting with integrin β1, and the increased number of ACs supported capillary formation and maturation.

In BBB–NETs, the CMFs in hydrogel are advantageous for brain capillary network formation and maturation. The CMFs increased the number of surviving ACs by interacting with integrin β1, and the increased number of ACs supported capillary formation and maturation (Figure 5), meaning that the effect of CMFs on astrocyte viability could be a metric in CMF quality control (QC). The QC of CMFs is important for producing a stable BBB–NET.

## Data Availability

Data are contained within the article.

## Supplementary Materials

The following supporting information can be downloaded at: https://www.mdpi.com/article/10.3390/biomedicines12112500/s1, Figure S1. Representative z-stack images of CD31 (a,b) and the IMARIS surface (c,d) in hydrogels with (b,d) and without (a,c) CMFs. Scale bar: 100 μm.

## Abbreviations

BBB	blood–brain barrier
MPS	microphysiological system
CNS	central nervous system
PKPD	pharmacokinetics/pharmacodynamics
TKTD	toxicokinetics/toxicodynamics
CoU	context of use
TfR	transferrin receptor
CMF	collagen microfiber
QC	quality control
EC	endothelial cell
AC	astrocyte
PC	pericyte
BM	basement membrane
ECM	extracellular matrix
SOP	standard operating procedure
