# Supplementary material for: Collagen I Microfiber Promotes Brain Capillary Network Formation in Three–Dimensional Blood–Brain Barrier Microphysiological Systems
Source: Biomedicines. 2024 Oct 31;12(11):2500. doi: 10.3390/biomedicines12112500 (PMC11591679; doi:10.3390/biomedicines12112500)
Supplement: Supplementary file 1 [file biomedicines-12-02500-s001.zip › biomedicines-3252028-supplementary.pdf]

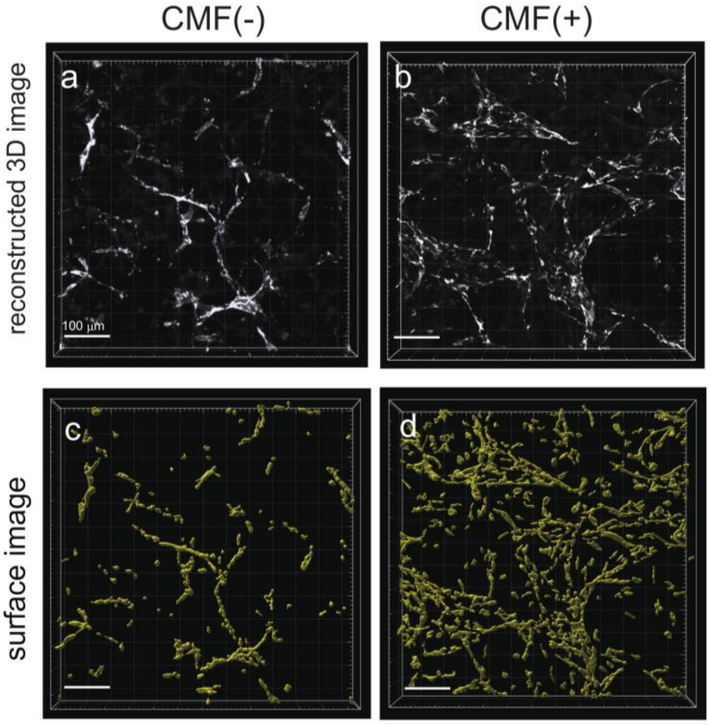

Figure S1. Representative z-stack images of CD31 (a,b) and the IMARIS surface (c,d) in hydrogels with (b,d) and without (a,c) CMFs. Scale bar: 100 μm
